# Placental SNV rs8192446 in the *ADORA2A* gene is associated with Preeclampsia: a pilot study

**DOI:** 10.3389/fphys.2026.1843298

**Published:** 2026-07-31

**Authors:** Perla Valenzuela-Torres, Martha Sosa-Macías, Carlos Felipe Burgos, Alicia E. Damiano, Mariano Reynoso, Luis E. Simental-Mendía, Jesenia Acurio, Laurence A. Marchat, Carlos Alonso Escudero, Carlos Galaviz-Hernandez

**Affiliations:** 1 National Polytechnic Institute (IPN), CIIDIR-Durango, Academia de Genómica, Durango, Mexico; 2 Universidad de Concepcion Departamento de Fisiologia, Concepción, Chile; 3 Departamento de Ciencias Biológicas, facultad de Farmacia y Bioquímica, Universidad de Buenos Aires, Buenos Aires, Argentina; 4 IFIBIO Houssay-CONICET Universidad de Buenos Aires, Buenos Aires, Argentina; 5 Instituto Mexicano del Seguro Social, Unidad de Investigación Durango, Durango, Mexico; 6 Vascular Physiology Laboratory, Department of Basic Science, Universidad del Bio Bio, Chillán, Chile; 7 Consortium for Research and Innovation Neurovascular, (NEUROVAS), Chillán, Chile; 8 Instituto Politecnico Nacional Escuela Nacional de Medicina y Homeopatia, Mexico City, Mexico

**Keywords:** *ADORA2A*, placenta, preeclampsia, rs8192446, SNV

## Abstract

**Introduction:**

Preeclampsia (PE) is one of the leading causes of maternal and fetal morbidity and mortality worldwide. An impaired trophoblast invasiveness and resulting defective remodeling of spiral arteries lead to placental hypoxia that promotes the release of different antiangiogenic and proinflammatory factors to maternal circulation. Adenosine a purine nucleoside derived from ATP metabolism, is among these factors, and high concentrations have been observed in the sera of women affected with PE. The raise in adenosine levels is linked to elevated placental expression of the adenosine A2A receptor (ADORA2A), yet the contribution of single-nucleotide variations (SNVs) in the *ADORA2A* gene remains unclear. The association between placental *ADORA2A* SNVs and preeclampsia was evaluated.

**Methods:**

Five SNVs (rs2298383, rs4822489, rs2236624, rs17650937, and s8192446) were genotyped using real-time PCR with TaqMan probes. The functional effect of the N144K mutation was evaluated through *in silico* mutagenesis to assess potential structural alterations.

**Results:**

The SNV rs8192446 was strongly associated with PE [OR=10.42 (95% CI: 2.08–103.39, p=0.0009)], association that remained after BH correction (p=0.0009). The SNV rs8192446 was also associated with mild PE (p=0.0403) and severe PE (p=0.0072). *In-silico* results revealed that N144K mutation does not alter the recognition of the ligand by *ADORA2A* but exerts a subtle modulation of the *ADORA2A*–adenosine interaction.

**Discussion:**

Taken together, these results suggest that placental *ADORA2A* rs8192446 is functionally relevant for preeclampsia development contributing to *ADORA2A* hypofunction through perturbations in downstream signaling mechanisms. This is the first study to report an association between *ADORA2A* rs8192446 and preeclampsia susceptibility in Mexican population.

## 1 Introduction

Preeclampsia (PE) is a hypertensive disorder of pregnancy that arises after 20 weeks of gestation and is a major contributor to maternal and fetal morbidity and mortality, especially in developing countries (American College of Obstetricians and Gynecologists (ACOG) and Task Force on Hypertension in Pregnancy, 2013). In Mexico, its prevalence ranges between 10% to 14%, making it one of the leading causes of maternal death, with an estimated 4,000 deaths annually (Barragán-Zúñiga et al., 2022). Globally, PE affects approximately 2–8% of pregnancies and significantly increases the risk of complications, including preterm birth, fetal growth restriction, and placental abruption (Roberts, 2024). Clinically, PE is characterized by new-onset hypertension and proteinuria, accompanied by systemic endothelial dysfunction. It is classified as mild (blood pressure ≥140/90 mmHg and proteinuria ≥300 mg/24 h) or severe (≥160/110 mmHg and proteinuria ≥3 g/24 h), and as early-onset (<34 weeks of gestation) or late-onset (≥34 weeks of gestation) (American College of Obstetricians and Gynecologists (ACOG) and Task Force on Hypertension in Pregnancy, 2013; WHO, 2016; Roberts, 2024).

The pathophysiology of PE involves defective trophoblastic invasion and poor remodeling of uterine spiral arteries, which result in placental hypoxia-reperfusion injury and the release of various anti-angiogenic and inflammatory mediators into maternal circulation (Roberts and Hubel, 2009; Yang et al., 2024). Among these factors, adenosine, a purine nucleoside derived from ATP metabolism, has been found elevated in maternal blood and placental tissue in PE (Salsoso et al., 2017), although its exact causes and consequences remain to be fully elucidated. Evidence suggests that adenosine is involved in regulating placental vascular development (Escudero et al., 2014; Iriyama et al., 2015; Acurio et al., 2017), modulating platelet function (Takeuchi et al., 2001; Macey et al., 2010), and may influence epigenetic processes such as DNA hypomethylation in the placenta, thereby affecting trophoblast differentiation, migration, and angiogenesis (Huang et al., 2017).

Adenosine acts via four G-protein-coupled receptors: A1, A2A, A2B, and A3. All these receptors have been implicated in angiogenesis and vascular regulation, including in the human placenta (Iriyama et al., 2015; Salsoso et al., 2017). Notably, the A2A receptor, encoded by the *ADORA2A* gene, has been shown to play a key role in endothelial cell function. Decreased A2A receptor expression or signaling is associated with reduced endothelial migration and proliferation in placental and umbilical cord endothelial cells (Acurio et al., 2017), suggesting its potential involvement in the vascular abnormalities of PE.

Furthermore, the study of Kurlak et al., in 2015 revealed that A2A receptor is strongly expressed in both the syncytiotrophoblast cells and the underlying cytotrophoblast particularly at early pregnancy stages (<20 weeks of gestation) showing a strong correlation with the expression of angiotensin IV receptor (AT4R) (Kurlak et al., 2015). This suggests an important relationship between A2A receptor and the renin-angiotensin system.

Another recent study evaluated the expression of the four different adenosine receptors between intrauterine growth restricted (IUGR) and normal placentas, revealing a significant decrease of A2A in IUGR and poor vascularized placentas, which was reversed after the treatment with adenosine. These changes were exerted by A2A that phosphorylates Stat3 and Akt increasing angiogenin-dependent angiogenesis (Wu et al., 2023b). The same group demonstrated that up regulation of A2A by adenosine supplementation improves placental vessel density in IUGR pigglets (Wu et al., 2023a).

The *ADORA2A* gene, located on chromosome 22q11.23, encodes a protein of 412 amino acids expressed in multiple tissues, including the placenta (NCBI Gene, 2024). Single nucleotide variants (SNVs) in this gene have been associated with several diseases. For example, rs2298383 has been reported to reduce transcription in patients with multiple chemical sensitivity (Cannata et al., 2020). The 1976 T/T genotype has been linked to increased systolic blood pressure following caffeine intake (Renda et al., 2012), and the rs4822489 has been associated with disease severity in Chinese patients with chronic heart failure (Zhai et al., 2015). Additionally, SNVs such as rs2236624 and rs4822489 have shown protective effects in patients with proliferative diabetic retinopathy (Charles et al., 2011). Missense variants in exon 3, including rs8192446 (Asn144Lys) and rs17650937 (Phe295Leu), were studied in patients with rheumatoid arthritis. However, because the non-polymorphic nature of rs8192446 and a minor allele frequency <5% of rs17650937 these variants were discarded (Hider et al., 2008).

Although the role of *ADORA2A* SNVs in PE has not been previously explored, their documented effects on vascular function, gene expression, and angiogenesis suggest a plausible link. Given that adenosine signaling is altered in PE and that A2A receptor dysfunction could compromise placental vascular adaptation, we hypothesize that placental SNVs in *ADORA2A* may be associated with susceptibility to this condition. Hence, this study aims to identify the potential association of placental SNVs in *ADORA2A* gene with diagnosis of PE.

## 2 Methods

### 2.1 Patient selection

A case-control study was carried out. Cases included placentas from women affected with PE and the control group included placentas from healthy pregnant women. All the participants consisted entirely of Mestizo women as self-referenced by the participants who declare at least two Mexican ascending generations. The diagnosis of PE (cases) was performed by a gynecologist according to the criteria of the American College of Obstetricians and Gynecologists (ACOG) (American College of Obstetricians and Gynecologists (ACOG) and Task Force on Hypertension in Pregnancy, 2013). Placentas from women classified as controls (healthy pregnant women- HPW) or women with PE (PE) were analyzed. Disease was further categorized according to the ACOG clinical criteria for preeclampsia, classifying cases as mild (blood pressure ≥140/90 mmHg and proteinuria ≥300 mg/24 h) or severe (≥160/110 mmHg and proteinuria ≥3 g/24 h) (American College of Obstetricians and Gynecologists (ACOG) and Task Force on Hypertension in Pregnancy, 2013), and these categories were subsequently used for stratified analyses. Where appropriate, comparisons were limited to individuals with valid genotypes for the involved SNVs.

The protocol was approved by the Ethics and Research Committee of the General Hospital of Durango (approval N. 596/022) in accordance with the Code of Ethics of the Declaration of Helsinki and its subsequent modifications. The patients decided to voluntarily participate in the study and signed the informed consent.

Pregnancies resolved by cesarean section were included. Patients with essential hypertension, impaired renal function, twin pregnancy, diabetes, recurrent spontaneous abortion, and placental abruption were excluded. Parameters such as systolic and diastolic blood pressure values, chronological age, gestational age, weight, height, body mass index, personal history of PE and gestational diabetes were recorded.

Human placentas were evaluated because they are the primary organ responsible for the origin and progression of preeclampsia. These tissues were obtained from HPW (n = 50) and preeclamptic (PE) (n = 50) pregnancies. The power of the study was calculated using the less common minor allele frequency of the studied SNVs [rs819244, (≈0.0047)]. *Post hoc* using the minor allele frequency (MAF) observed in the studied groups (HPW 3%; PE 18%), revealed that power obtained was approximately 89%, adequate for the observed effect.

### 2.2 Collection of placental tissue

After delivery of the placenta (collection time no longer than 30 min), and removal of decidua, 5 × 5 mm biopsies were obtained from the cotyledons on the maternal side. Twelve biopsies were taken, three from each placenta quadrant and washed in 4°C cold 1x phosphate-buffered saline (PBS; in mmol/L: 130 NaCl, 2.7 KCl, 0.8 Na_2_HPO_4_, 1.4 KH_2_PO_4_, 4°C, pH 7.4) to eliminate blood traces. The sample was then immersed in RNAlater 1:1 (Sigma-Aldrich™) and stored at −80 °C for further DNA/RNA and protein extraction.

### 2.3 DNA extraction and genotyping analysis

DNA was extracted from placental tissue following the modified methodology of Miller and Gilbert (2001) (Miller et al., 1988). DNA integrity and concentration were verified by 1% agarose gel electrophoresis and spectrophotometry on a NanoDrop 2000™ (Thermo Fisher, Waltham, MA, USA), respectively.

Genotyping analysis for five single nucleotide variants (SNVs): rs2298383, rs4822489, rs2236624, rs8192446 and rs17650937 in the *ADORA2A* gene was performed using IDT probes: Hs.GT.rs2298383.T.1, Hs.GT.rs4822489.G1, Hs.GT.rs2236624.C1, CD.GT.SKJL8070.1 and CD.GT.XZPV8924.1 respectively on StepOne™ Quantitative Real-Time PCR (qPCR) System (Applied Biosystems^®^, Carlsbad, CA, USA). The PCR reaction conditions consisted of an initial denaturation step at 95 °C for 10 minutes, followed by 42 cycles of denaturation at 95 °C for 15 seconds, annealing at 60 °C for 1 minute, and extension at 60 °C for 1 minute.

### 2.4 *In silico* prediction of A2AR wild type and mutant N144K

#### 2.4.1 Structure preparation

The *in silico* predictions were performed using the available high-resolution (2.71 Å) crystal structure of the human A2A receptor bound to a synthetic agonist, obtained through X-ray crystallography (PDB ID: 3QAK) (Xu et al., 2011).

The structure was optimized using the Protein Preparation Wizard module of Maestro (Schrödinger 2024, LLC, NY) including bond order assignment, addition of missing hydrogen, optimization of protonation states and side-chain orientations. The optimization was completed by energy minimization using a Polak-Ribière conjugate gradient with MacroModel (Schrödinger 2024, LLC, NY), defining a convergence threshold of 0.05 kcal/mol x Å.

To evaluate the effect of the SNV rs8192446 on the receptor’s structure and stability, the Asn144Lys mutation was generated using the Residue and Loop Mutation module of Maestro (Schrödinger 2024, LLC, NY) followed by energy minimization within a 5 Å radius of the mutation site in an implicit solvent environment.

#### 2.4.2 Molecular dynamics

Molecular dynamics simulations for both the wild-type and mutant N144K A2ARs were performed using Desmond (Schrödinger 2024, LLC, NY). All systems were individually constructed and placed in an orthorhombic box with a radius of 10 Å, centered on a 1-palmitoyl-2-oleoylphosphatidylcholine (POPC) lipid bilayer, and then solvated with a single point charge (SPC) water model. Na^+^ and Cl^-^ ions were added to both sides of the membrane at a concentration of 0.15 M. Subsequently, the system was equilibrated under 5 kcal·mol^-^¹·Å^-^² constraints for 20 ns for receptors in the apo (ligand-free) state and for 100 ns for protein-ligand complexes, using an NPγT (constant normal pressure, constant membrane lateral surface tension, and constant temperature [CE5.1]) set at 300 K and 1 bar pressure. Finally, molecular dynamics (MD) simulations of 250 ns were performed for each of the systems. The results are analyzed according to the root mean square deviation (RMSD) of the free protein and the protein-ligand, as well as the root mean square fluctuation (RMSF) values ​​of the protein structure and the protein-ligand interaction fractions calculated by Desmond (Schrödinger 2024, LLC, NY).

#### 2.4.3 Protein-ligand docking

To evaluate the effect of the N144K mutation on the receptor’s ability to interact with its agonist, protein–ligand docking predictions were performed using the structure of A2AR (PDB ID: 3QAK), with both the wild-type and N144K mutant receptor model. Initially, receptor grids were generated and centered on the ligand-binding site defined by the co-crystallized agonist in the reference structure. Docking calculations using adenosine (CID: 60961) were performed using Glide (Schrödinger 2024, LLC, NY) in Extra Precision (XP) mode, a high-accuracy docking protocol designed to provide a balanced compromise between sampling exhaustiveness and computational efficiency. For all generated complexes, energetic parameters were calculated using Prime (Schrödinger, LLC, NY). The resulting protein–ligand complexes were evaluated based on their structural and energetic descriptors, including the docking score and the theoretical binding free energy (ΔG_bind_) calculated using the Molecular Mechanics–Generalized Born Surface Area (MM-GBSA) approach. All figures were generated using Maestro.

### 2.5 Statistical analysis

Continuous variables are presented as mean ± standard deviation, count variables as median [interquartile range], and categorical variables as n (%). Between-group differences in continuous variables were assessed using the independent-samples Student’s t-test, whereas count variables were compared using the Wilcoxon rank-sum test. Categorical variables were compared using Fisher’s exact test or Pearson’s chi-square test, as appropriate. Normality of continuous variables was evaluated using the Shapiro–Wilk test, and homogeneity of variances was assessed using Levene’s test. Genotype frequencies were obtained by direct counting, allele frequencies were calculated, and Hardy-Weinberg equilibrium (HWE) was obtained using the exact test. The association between SNV and PE was determined by Firth penalized logistic regression models in all genetic association analyses. Cohen’s d analysis was used as a standardized measure of effect size derived from odds ratios, allowing the magnitude of the association between genetic variants and PE to be quantified. Linkage disequilibrium (LD) was calculated (D, D’, r, and r²). Haplotype analysis between rs17650937 and rs8192446 was calculated, using the expectation–maximization (EM) algorithm (haplo.em) for haplotype reconstruction and the haplo.score function to assess the association between haplotypes and PE under an additive binomial model. Power analyses (*a priori* and *post hoc*) were done by comparing two proportions with unequal sample sizes (pwr.2p2n.test and ES.h), using both the reported population allele frequency and that observed in the studied cohort. In all cases, the p-values derived from homogeneous families of hypotheses were adjusted by false discovery rate (FDR) control using the Benjamini-Hochberg (BH) method. All statistical analyses were performed in R using the RStudio 2025.09.2 + 418 “Cucumberleaf Sunflower” Release interface (Windows 11, 64-bit) and reproducible documents generated with Quarto 1.7.32. Further information regarding statistical analysis was integrated as **Supplementary Methods** (SM1).

## 3 Results

### 3.1 Patients

The maternal, obstetric, and neonatal characteristics of the study population are summarized in **Table 1**. Significant differences between the PE and HPW groups were observed in the distribution of previous pregnancies, systolic and diastolic blood pressure, gestational age at delivery, and birthweight. The distribution of previous pregnancies differed significantly between groups [2 (1–2) in PE vs. 2 (2–4) in HPW, p = 0.0153]. Women with PE showed markedly higher systolic and diastolic blood pressure values (both p < 0.0001) and delivered at an earlier gestational age than HPW (35.2 ± 3.3 vs. 38.3 ± 1.5 weeks, p < 0.0001). Birthweight was also significantly lower in the PE group for both female (2343.1 ± 772.1 vs. 3281.4 ± 465.3 g, p = 0.0070) and male newborns (2613.9 ± 749.8 vs. 3460.1 ± 386.9 g, p = 0.0001).

**Table 1:** Maternal, obstetric, and neonatal characteristics of the study population.

Variable		PE n=50	HPW n=50	*p*
Age (years)		27.6 ± 8.0	26.9 ± 6.6	0.4472
Weight (kg)		77.5 ± 21.2	72.2 ± 12.2	0.6761
Height (cm)		151.4 ± 34.5	160.1 ± 6.3	0.0513
BMI (kg/m^2^)		30.6 ± 8.7	28.2 ± 4.8	0.8210
Obesity (n, %)		23 (46.0)	17 (34.0)	0.0624
Previous pregnancies		2 [1–2]	2 [2–4]	0.0153
Previous deliveries		0 [0–1]	1 [0–2]	0.0593
Previous cesarean deliveries		1 [0–1]	0 [0–1]	0.1401
Previous abortions		0 [0–0]	0 [0–0]	0.7233
History of PE (n, %)		5 (10.0)	2 (4.0)	0.4326
History of GDM (n, %)		3 (6.0)	0 (0.0)	0.2412
SBP (mmHg)		157.2 ± 3.2	112.8 ± 12.3	<0.0001
DBP (mmHg)		91.6 ± 3.7	72.8 ± 9.9	<0.0001
Gestational age at diagnostic (weeks)		34.4 ± 3.4	—	—
Gestational age at delivery (weeks)		35.2 ± 3.3	38.3 ± 1.5	<0.0001
Fetal sex (n, %)	Female	19 (38.0)	16 (32.0)	0.7866
	Male	31 (62.0)	34 (68.0)	
Birthweight (g)	Female	2343.1 ± 772.1	3281.4 ± 465.3	0.0070
	Male	2613.9 ± 749.8	3460.1 ± 386.9	0.0001

Data are presented as mean ± SD for continuous variables, median [IQR] for count variables, and as n (%) for categorical variables. Between-group comparisons were performed using the Student t test for continuous, Wilcoxon rank-sum test for count variables and Fisher’s exact or Chi-square test for categorical variables. Obesity was defined as body mass index ≥30 kg/m². Birthweight was analyzed separately according to fetal sex; the corresponding p-values compare PE and HPW within each fetal-sex stratum. All p-values are two-sided. PE, preeclampsia; HPW, healthy pregnant women; BMI, body mass index; GDM, gestational diabetes mellitus; SBP, systolic blood pressure; DBP, diastolic blood pressure; SD, standard deviation; IQR, interquartile range.

No significant between-group differences were found in maternal age, weight, height, BMI, obesity prevalence, previous deliveries, previous cesarean deliveries, previous abortions, history of PE, history of gestational diabetes mellitus, or fetal sex distribution (all p > 0.05).

Women with PE were further divided according to mild (MiPE) or severe (SePE) preeclampsia (**Table 2**). Significant differences between the severe and mild PE groups were observed only for systolic and diastolic blood pressure. Systolic blood pressure was 149.2 ± 6.9 mmHg in MiPE and 173.5 ± 15.9 mmHg in SePE (p = 0.0001), whereas diastolic blood pressure was 92.1 ± 21.2 and 108.9 ± 18.3 mmHg, respectively (p = 0.0072).

**Table 2:** Maternal, obstetric, and neonatal characteristics according to severity of preeclampsia.

Variable		MiPE n=25	SePE n=25	*p*
Age (years)		25.5 ± 8.0	29.9 ± 7.6	0.0632
Weight (kg)		73.6 ± 12	81.7 ± 28	0.1411
Height (cm)		159.9 ± 5.2	142.4 ± 4.8	0.0997
BMI (kg/m^2^)		28.8 ± 4.9	34.7 ± 8.3	0.0667
Obesity (n, %)		13 (52.0)	10 (40.0)	0.0724
Previous pregnancies		1 [1–2]	2 [1–4]	0.3643
Previous deliveries		0 [0–1]	0 [0–1]	0.3532
Previous cesarean deliveries		1 [0–1]	1 [0–1]	0.4692
Previous abortions		0 [0–0]	0 [0–0]	0.4309
History of PE (n, %)		1 (4.0)	4 (16.0)	0.1745
History of GDM (n, %)		2 (8.0)	1 (4.0)	0.9001
SBP (mmHg)		149.2 ± 6.9	173.5 ± 15.9	0.0001
DBP (mmHg)		92.1 ± 21.2	108.9 ± 18.3	0.0072
Gestational age at diagnostic (weeks)		35.0 ± 3.0	33.5 ± 3.5	0.4376
Gestational age at delivery (weeks)		35.1 ± 3.1	34.0 ± 8.1	0.6913
Fetal sex (n, %)	Female	9 (36.0)	10 (40.0)	0.9754
	Male	16 (64.0)	15 (60.0)	
Birthweight (g)	Female	2212.9 ± 613.3	2489.6 ± 941.4	0.4786
	Male	2430.0 ± 730.0	2811.9 ± 747.7	0.1914

Data are presented as mean ± SD for continuous variables, median [IQR] for count variables, and as n (%) for categorical variables. Between-group comparisons were performed using the Student t test for continuous, Wilcoxon rank-sum test for count variables and Fisher’s exact or Chi-square test for categorical variables. Obesity was defined as body mass index ≥30 kg/m². Birthweight was analyzed separately according to fetal sex; the corresponding p-values compare MiPE and SePE within each fetal-sex stratum. All p-values are two-sided. MiPE, mild PE; SePE, severe PE; PE, preeclampsia; BMI, body mass index; GDM, gestational diabetes mellitus; SBP, systolic blood pressure; DBP, diastolic blood pressure; SD, standard deviation; IQR, interquartile range.

No significant differences were observed in maternal age, weight, height, BMI, obesity prevalence, previous pregnancies, previous deliveries, previous cesarean deliveries, previous abortions, history of PE, history of gestational diabetes mellitus, gestational age at diagnosis, gestational age at delivery, fetal sex distribution, or birthweight stratified by fetal sex (all p > 0.05).

### 3.2 Analysis of SNVs association with PE

The five SNVs were analyzed for their potential association with PE. For the first three SNVs (rs2298383, rs4822489, and rs2236624), no associations were observed between genotypes and PE. For rs17650937, the CT genotype was negatively associated with PE, suggesting a potential protective effect [OR=0.33 (95% CI: 0.12–0.92, p=0.0234)]. Conversely, TT genotype was associated with PE [OR=2.99 (95% CI: 1.08–8.64, p=0.0234)]. Both genotypes remained significant after BH correction within the SNV. The minor allele C showed no association with PE (**Table 3**).

**Table 3:** Association of genotypes and alleles with PE.

SNV	Genotypes alleles	HPW	PE	OR [95% CI]	p*	*p* ^§^
rs2298383	AA	11 (0.26)	9 (0.20)	0.73 [0.23 to 2.22]	0.6135	0.6135
	CA	17 (0.40)	25 (0.57)	1.92 [0.76 to 4.99]	0.1390	0.4171
	CC	14 (0.33)	10 (0.22)	0.59 [0.20 to 1.69]	0.3389	0.5083
	A	39 (0.46)	43 (0.49)	1.10 [0.59 to 2.08]	0.7624	0.7624
	C	45 (0.54)	45 (0.61)			
rs4822489	AA	13 (0.31)	10 (0.23)	0.66 [0.22 to 1.90]	0.4680	0.4680
	CA	29 (0.69)	34 (0.77)	1.52 [0.52 to 4.50]	0.4680	0.4680
	A	55 (0.65)	54 (0.61)	1.19 [0.61 to 2.33]	0.6360	0.7624
	C	29 (0.35)	34 (0.39)			
rs2236624	CC	2 (0.05)	3 (0.07)	1.46 [0.16 to 18.30]	1.0000	1.0000
	CT	19 (0.45)	23 (0.52)	1.32 [0.52 to 3.37]	0.5268	0.7903
	TT	21 (0.50)	18 (0.41)	0.70 [0.27 to 1.77]	0.5161	0.7903
	C	23 (0.27)	29 (0.33)	1.30 [0.65 to 2.63]	0.5070	0.7624
	T	61 (0.73)	59 (0.67)			
rs17650937	TT	18 (0.43)	25 (0.69)	2.99 [1.08 to 8.64]	0.0234	0.0234
	CT	24 (0.57)	11 (0.31)	0.33 [0.12 to 0.92]	0.0234	0.0234
	T	60 (0.71)	61 (0.85)	0.45 [0.18 to 1.06]	0.0554	0.1384
	C	24 (0.29)	11 (0.15)			
rs8192446	GG	38 (0.95)	23 (0.64)	0.09 [0.01 to 0.48]	0.0009	0.0009
	CG	2 (0.05)	13 (0.36)	10.42 [2.08 to 103.39]	0.0009	0.0009
	G	78 (0.97)	59 (0.82)	8.33 [1.82 to 100.00]	0.0018	0.0091
	C	2 (0.03)	13 (0.18)			

*Fisher exact test, ^§^Benjamini-Hochberg (BH) correction.

The analysis of SNV rs8192446, showed that the CG genotype was strongly associated with PE [OR=10.42 (95% CI: 2.08–103.39, p=0.0009)], association that remained after BH correction (p=0.0009). On the contrary, the GG genotype manifest a potential protective effect [OR=0.09 (95% CI: 0.01–0.48, p = 0.0009)], which remained after BH correction (p=0.0009). The analysis of alleles revealed a potential risk effect of minor allele C [OR=8.33 (95% CI: 1.82–100.00, p = 0.0018)], remaining after BH correction (p=0.0091) (**Table 3**).

The results of HWE analysis in controls revealed that rs2298383 (p=0.2275), rs2236624 (p=0.6960) and rs8192446 (p=1.000) are in equilibrium, meanwhile rs4822489 (p=0.0005) and rs17650937 (p=0.0180) are not.

When considering inheritance models, rs2298383, rs4822489, rs2236624, and rs17650937 showed no significant association with PE. In contrast, rs8192446 demonstrated a strong association with PE under a dominant inheritance model [OR=25.07 (95% CI: 2.93–1107.33, p=0.0016)], that persisted after BH correction (p=0.0078). This pattern is consistent with the results of the dominant and genotypic models, indicating a strong association between this variant and PE risk (**Table 4**). Both, dominant and additive models behave similarly because of the absence of homozygotes for the minor allele.

**Table 4:** Association analysis based on inheritance models.

SNV	Inheritance model	n	OR(a) [95% CI]	Cohen *d*	p*	*p^§^*
rs2298383	Dominant	80	1.17 [0.38 to 3.62]	0.09	0.7872	0.7872
	Recessive		0.93 [0.26 to 3.24]]	-0.04	0.9065	0.9065
	Codominant AA vs AC		0.83 [0.21 to 3.22]	-0.10	0.7891	0.7891
	Codominant CC vs AC		0.80 [0.24 to 2.69]	-0.12	0.7127	0.7891
	Additive		1.04 [0.51 to 2.12]	0.02	0.9190	0.9190
rs4822489	Dominant	80	1.57 [0.49 to 5.30]	0.25	0.4494	0.7490
	Additive		1.57 [0.49 to 5.30]	0.25	0.4492	0.9190
rs2236624	Dominant	80	1.20 [0.40 to 3.53]	0.10	0.7432	0.7872
	Recessive		2.75 [0.36 to 22.80]	0.56	0.3133	0.9065
	Codominant CC vs CT		2.65 [0.32 to 23.59]	0.54	0.3526	0.7052
			0.96 [0.31 to 3.02]	-0.02	0.9377	0.7052
	Codominant TT vs CT					
	Additive		1.34 [0.56 to 3.23]	0.16	0.5042	0.9190
rs17650937	Dominant	72	0.54 [0.17 to 1.72]	-0.34	0.2962	0.7406
	Additive		0.54 [0.17 to 1.71]	-0.34	0.2962	0.9190
rs8192446	Dominant	70	25.07 [2.93 to 1107.33]	1.78	0.0016	0.0078
	Additive		25.07 [2.93 to 1107.33]	1.78	0.0016	0.9190

* Logistic regression with Firth correction, ^§^Benjamini-Hochberg (BH) correction. Covariates used: BMI, previous pregnancies, previous cesarean sections, previous deliveries, previous abortions, history of PE and history of GDM.

When stratifying PE according to severity, differential patterns were observed between rs17650937 and rs8192446. For rs17650937, carriers of the minor allele had a lower risk of MiPE compared to controls (p=0.0059). No significant association was observed with SePE. When comparing SePE versus MiPE the former showed a marked reduction in the frequency of the minor allele, consistent with a possible protective effect of rs17650937 against progression to more severe forms of PE. Regarding rs8192446, carrying the minor allele was associated with an increased risk of MiPE (p=0.0403). The risk was even higher for SePE (p=0.0072). The contrast between SePE and MiPE showed no association with the progression of the disease to a more severe form (p=0.1972) (**Table 5**). Overall, the analysis indicates that rs17650937 may have a protective role against MiPE, while rs8192446 behaves as a robust risk factor (at least in our sample) especially in the more severe forms.

**Table 5:** Analysis of association according to PE severity.

SNV	Contrast	Covariates	n	OR(a) [95% CI]	p*
rs17650937	MiPE vs Control	7	60	0.08 [0.03 to 0.53]	0.0059
	SePE vs Control	6	59	2.11 [0.45 to 12.30]	0.3442
	SePE vs MiPE	7	37	51.75 [4.35 to 2337.90]	0.0001
rs8192446	MiPE vs Control	7	56	13.67 [1.12 to 430.79]	0.0403
	SePE vs Control	7	59	20.22 [2.20 to 302.93]	0.0072
	SePE vs MiPE	7	37	4.04 [0.49 to 53.12]	0.1972

Covariates used: 6 = BMI, previous pregnancies, previous cesarean sections, previous deliveries, previous abortions, history of PE; 7 = 6 + history of GDM. * Logistic regression with Firth correction.

### 3.3 Analysis of haplotypes and their association with PE

The analysis of haplotypes revealed a strong linkage disequilibrium between rs17650937 and rs8192446 (D´=0.9970). However, their low correlation (r^2^ = 0.0268) suggests that one of the alleles cannot predict the other one. This reinforces the previous results that showed independent or complementary association for both SNVs. Two haplotypes (CG and TC) demonstrated specific contributions as shown in **Table 6**.

**Table 6:** Haplotypes association with PE.

Haplotype	Frequency	Score	p*	*p^§^*
TG (ref)	0.6875	0.00	1.0000	1.0000
CG	0.2292	-2.22	0.0263	0.0395
TC	0.0833	2.97	0.0030	0.0089

* Haplotype score tests, ^§^Benjamini-Hochberg (BH) correction.

TC haplotype showed a significant positive association with PE, suggesting that this specific allele combination may increase susceptibility to the disease. Conversely, the CG haplotype exhibited a negative score and a p-value consistent with a higher prevalence in controls, which could be interpreted as a possible protective pattern at an exploratory level.

### 3.4 Functional effect of the N144K mutation

**Figure 1:**
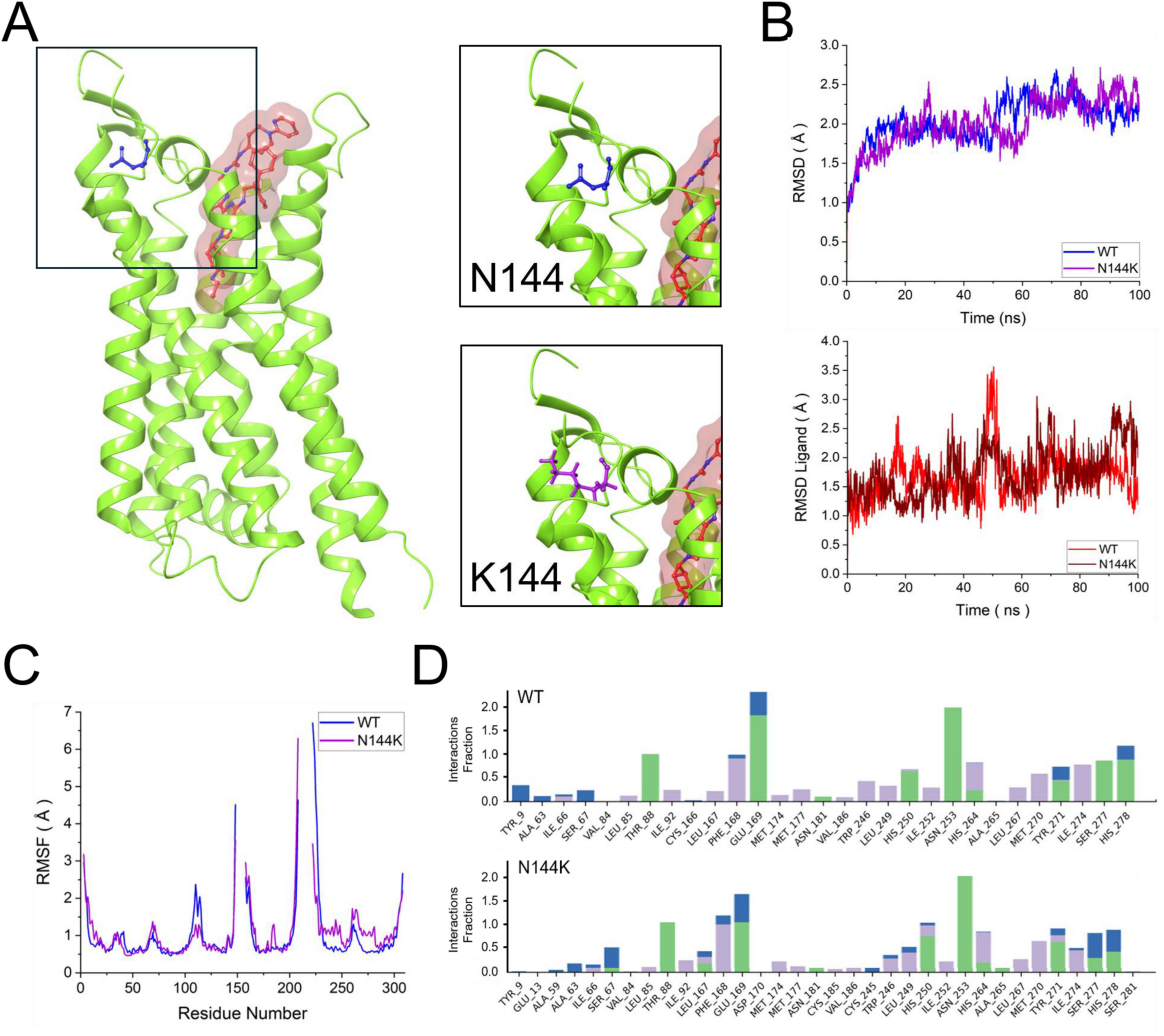
Predictive analysis of the effect of the N144K mutation on A2A receptor structure and activation. **(A)** *In silico* mutagenesis used to generate the N144K mutant. Insets show a zoomed view of residue 144, highlighting the corresponding side chains. The agonist molecule (UK-432097) is shown in red, with its molecular surface representation. **(B)** RMSD trajectories of the receptor (top) and ligand (bottom) during molecular dynamics simulations of the wild-type and N144K mutant A2A receptors. **(C)** Comparison of RMSF profiles calculated for the wild-type and N144K mutant A2A receptors. The structural model contains two gaps spanning residues 149–157 and 209–221. **(D)** Interaction profiles between the agonist and both wild-type and N144K mutant A2A receptors. Colors indicate interaction types as follows: hydrogen bonds (green), hydrophobic interactions (light purple), and water-mediated hydrogen bonds (blue). Bar height represents the fraction of the simulation time during which each interaction is detected.

To predict whether the N144K mutation identified in genetic studies affects the structure of the A2AR, *in silico* mutagenesis was performed to assess potential structural alterations. A residue-centered comparison at position 144 revealed that substitution with lysine induces only local changes that could affect the agonist binding site due to its structural proximity (**Figure 1A**). Given that initial energy minimization is restricted to the vicinity of the mutation, molecular dynamics (MD) simulations were subsequently conducted for both the wild-type and N144K mutant A2ARs to evaluate their global structural stability.

Analysis of root mean square deviation (RMSD) values, as an indicator of structural variation along the simulation, showed that both systems remained below 3 Å, indicating structural stability throughout the 100 ns trajectories and supporting that the mutation does not destabilize the A2AR (**Figure 1B**). Consistently, agonist binding was preserved, with discrete conformational changes observed during the simulations (**Figure 1B**). A2AR–agonist complexes exhibited minimal structural fluctuations, as evidenced by RMSF profiles, where the mutant displayed only minor deviations compared to the wild-type receptor (**Figure 1C**). The interaction profiles indicate that agonist binding is largely conserved between the wild-type and N144K A2A receptors, with key contacts involving Thr88, Asn253, His250, His264, Tyr271, Ser277, and His278 remaining stable throughout the simulations. The N144K variant exhibits an increased contribution of Glu169, particularly through water-mediated hydrogen bonds, suggesting a local reorganization of the hydrogen-bonding network surrounding the ligand. Additional increases in interaction occupancy are observed for Tyr271 and His278, whereas contacts involving residues such as Tyr9, Phe168, and Trp246 are reduced relative to the wild-type receptor. Furthermore, low-frequency interactions with residues including Asp170, Cys245, and Ser281 emerge exclusively in the mutant complex, indicating a redistribution of ligand–receptor contacts (**Figure 1D**; **Supplementary Figure 1**). Collectively, these findings suggest that the N144K substitution does not alter the structural integrity of the orthosteric binding pocket or compromise agonist binding; rather, it induces subtle rearrangements in the local interaction network that may influence receptor conformational dynamics and downstream activation mechanisms.

**Figure 2:**
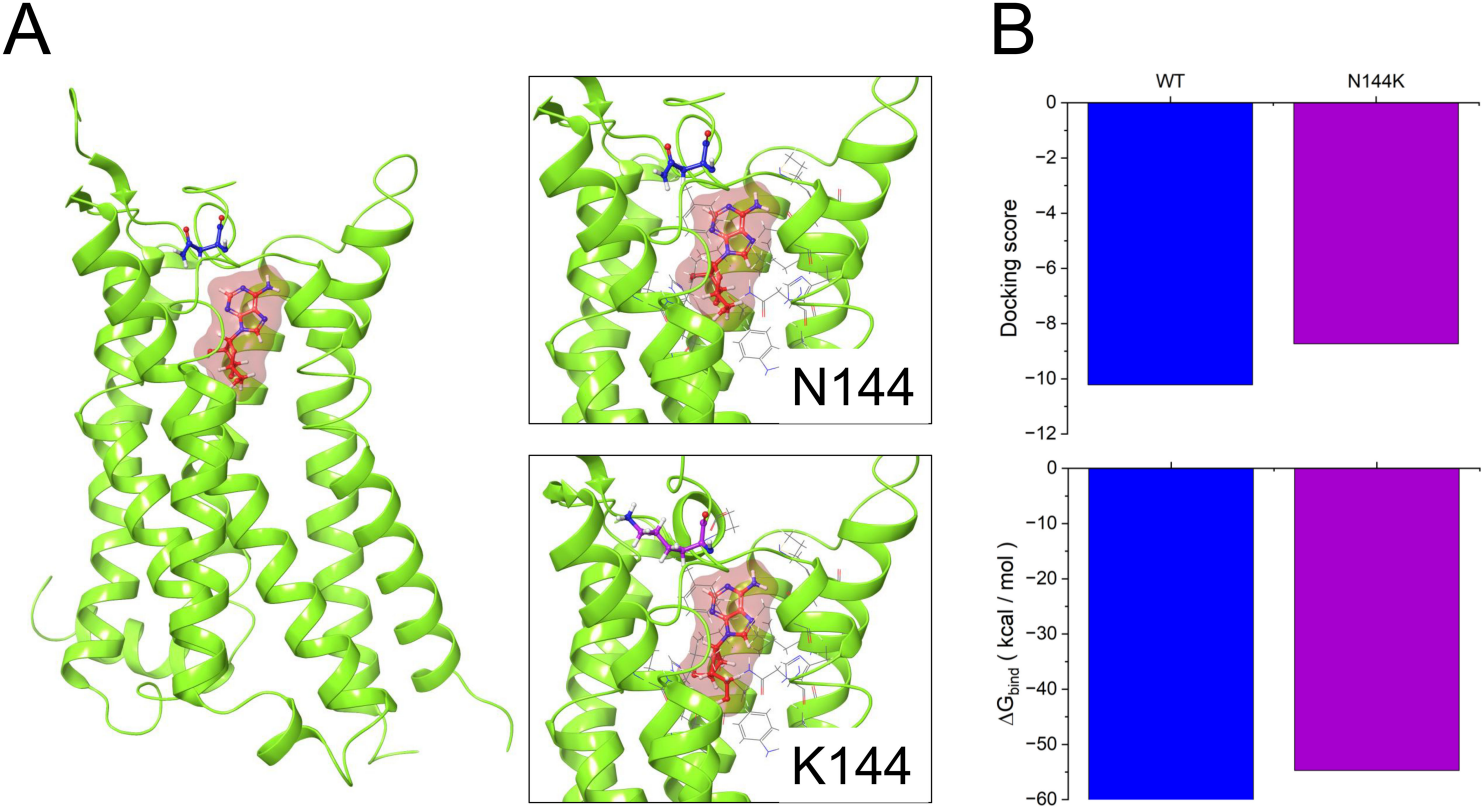
Predicted interaction between A2AR and adenosine based on protein–ligand docking. **(A)** The top-ranked poses selected for the A2AR (green)–adenosine (red) complexes are shown for both the wild-type and N144K mutant receptors. The agonist is depicted with its molecular surface representation. **(B)** Residues N144 and K144 are displayed with their side chains in a sticks representation and are colored blue and magenta, respectively.

Given the absence of major structural alterations in the agonist-binding pocket, we next evaluated whether the N144K mutation influences the interaction between A2AR and its endogenous agonist, adenosine. To this end, protein–ligand docking predictions were performed using adenosine and both the wild-type and N144K mutant receptor models. Stable receptor–ligand complexes were obtained in both cases, with the agonist adopting a conserved binding pose within the orthosteric site. Nevertheless, subtle differences in the interaction network were observed in the mutant receptor, resulting in a slight modification of the predicted binding affinity. Specifically, the docking score changed from -10.221 to -8.734 kcal/mol, while the theoretical binding free energy (ΔG_bind_) shifted from -60.51 to -54.74 kcal/mol (**Figure 2**).

Considering previously reported functional alterations in PE, these findings support the hypothesis that the N144K mutation may contribute to A2AR hypofunction through perturbations in downstream signaling mechanisms, rather than through impaired agonist binding.

## 4 Discussion

The most relevant finding of this study was the consistent association of the *ADORA2A* gene rs8192446 gene variation with preeclampsia. In the genotypic analysis, the CG genotype was associated with a significantly increased risk of developing preeclampsia, while the GG genotype showed a possible protective effect. Furthermore, under the dominant model, rs8192446 maintained an association with the disease even after Benjamini-Hochberg correction. The consistency between the genotypic and dominant models suggests that this variant represents the strongest genetic signal in our sample. This result is particularly significant because rs8192446 was found in Hardy-Weinberg equilibrium in the controls, providing internal support for the validity of the finding, unlike other loci in the panel whose interpretation requires more caution (Wigginton et al., 2005; Namipashaki et al., 2015). This association is supported by the involvement of the adenosine-A2AR pathway in the placental pathophysiology of preeclampsia. Placental expression of adenosine receptors, including A2A, has been shown to be increased in placentas from pregnancies with preeclampsia, and hypoxia specifically increases A2A receptor expression, suggesting an adaptive response to impaired placental perfusion (von Versen-Höynck et al., 2009; Kurlak et al., 2015).

Additionally, elevated adenosine concentrations have been described in both maternal and fetal circulation in preeclampsia, and this increase is associated with greater clinical severity, chronic uteroplacental ischemia, and immunological alterations such as Th1/Th2 imbalance (Yoneyama et al., 2002a; Yoneyama et al., 2002b; Espinoza et al., 2011). Taken together, this evidence supports the idea that genetic variation in *ADORA2A* could modulate the placental response to hypoxia, inflammation, and vascular dysfunction—central processes in the disease.

When preeclampsia was stratified by degree of severity, differential patterns were observed between rs17650937 and rs8192446. While rs8192446 maintained a behavior consistent with a risk factor, rs17650937 showed a possible protective role against mild preeclampsia. This difference is biologically reasonable considering that SePE and MiPE do not represent identical phenotypes: SePE is usually associated with an early onset preeclampsia (EOPE), greater placental injury, maternal poor perfusion, and worse maternal-fetal outcomes, while MiPE usually appearing as late onset preeclampsia (LOPE), shows more marked maternal involvement and less severe placental alteration (Kovo et al., 2012; Teka et al., 2023).

Accordingly, the A2AAR/NO/VEGF pathway has been described as exhibiting different behaviors depending on the clinical subtype: in EOPE, there is lower A2AAR expression, lower NO and VEGF synthesis, and reduced angiogenic capacity, while in LOPE, the pathway retains a relatively more pronounced proangiogenic effect (Escudero et al., 2013). Furthermore, A2AAR regulates VEGF expression in the fetoplacental endothelium of normal pregnancies and those with late-onset preeclampsia (Acurio et al., 2017).

Therefore, the differential association observed between these two SNPs may reflect a subtype-specific modulation of the ADORA2A-dependent angiogenic and adaptive response (Kovo et al., 2012; Escudero et al., 2013; Acurio et al., 2017; Teka et al., 2023).

Haplotype analysis helped to better understand the relationship between rs17650937 and rs8192446. Although both SNPs showed very high linkage disequilibrium (D′=0.9970), the correlation between them was very low (r²=0.0268). This means that, although there has been almost no recombination between these variants over time, knowing one of them does not allow for good prediction of the other. This behavior can be explained by the fact that the r² value is highly dependent on allele frequency and therefore can be low even when D′ is very high (VanLiere and Rosenberg, 2008).

Based on this, the fact that the TC haplotype was positively associated with preeclampsia and that the CG haplotype appeared more frequently in controls suggests that the genetic risk within *ADORA2A* could be better explained by certain allele combinations than by a single SNV. However, due to the low r² value, these results should be interpreted with caution and understood more as a sign that the haplotype structure might be important, rather than as evidence that one marker can replace the other (VanLiere and Rosenberg, 2008).

The in-silico results allow us to link the genetic association with a possible functional consequence. The fact that the N144K mutation did not destabilize the A2AR receptor and preserved agonist binding suggests that this variant would not significantly alter orthosteric recognition or the overall stability of the receptor. However, the prediction of a subtle modulation of the A2AR–adenosine interaction, with an increase in estimated affinity, suggests that the functional effect could be in later stages of signal transduction. This interpretation is consistent with structural studies demonstrating that A2AR is a Gs-coupled receptor whose signaling efficiency depends on a complex network of conformational states during G protein coupling; therefore, discrete changes can modify the productivity of the complex without necessarily altering agonist binding (Olah, 1997; Huang et al., 2021). Consequently, these findings support the hypothesis that N144K could contribute to a functional alteration of A2AR at the post-receptor level, rather than through a primary ligand-binding defect (Olah, 1997; Huang et al., 2021).

Taken together, our results suggest that *ADORA2A* may be involved in genetic susceptibility to preeclampsia through mechanisms related to placental hypoxia, angiogenesis, and A2AR receptor signaling efficiency. Within this framework, rs8192446 emerges as the most important signal in the sample, supported by the convergence between inheritance models, its persistence in HWE in controls, and the biological plausibility of the adenosinergic pathway in the placenta, however this result must be considered as preliminary. Meanwhile, rs17650937 and the identified haplotypes provide clues to a more complex genetic architecture. A couple of SNVs were observed in HWD which may be related to the relatively small sample size and the distribution of allele frequencies in the studied population. However, they do not affect the main result.

Some limitations in contextualizing these findings are the small sample size, which is the result of very strict selection criteria, resulting in high width of some confidence intervals. It is clear that wide ranges imply that population is small but do not discard the results a-priori so, these results must be considered as preliminary and stimulate us to increase the sample size in a future study evaluating also a higher number of SNVs.

Despite its importance, the observed *in-silico* association must be taken cautiously. Therefore, it will be necessary to replicate these results in independent cohorts and complement them with *in-vitro* functional studies, including the integration of placental ADORA2A gene/protein expression assays (currently under investigation) that allow us to define whether these variants modify the expression, coupling or effective signaling of A2AR in preeclampsia.

As a conclusion, the study of this placental rs8192446 is preliminary and must be considered as a precursor of further studies evaluating women and men to be considered as a potential predictive marker.

## Data Availability

The datasets presented in this study can be found in online repositories. The names of the repository/repositories and accession number(s) can be found in the article/**Supplementary Material**.

## Supplementary material

Supplementary Figure 1 Protein–ligand interaction diagrams of A2AR complexes with the agonist UK-432097. Interactions within a 4 Å radius of the agonist are shown for both wild-type and N144K mutant A2A receptors. Amino acids are colored according to their physicochemical properties: polar (cyan), hydrophobic (green), negatively charged (red), and water molecules (white). Interaction types are represented as follows: hydrogen bonds (magenta arrows), π–π stacking (green lines), and solvent-exposed regions (gray halos).
