# Supplementary material for: Placental SNV rs8192446 in the ADORA2A gene is associated with Preeclampsia: a pilot study
Source: Front Physiol. 2026 Jul 31;17:1843298. doi: 10.3389/fphys.2026.1843298 (PMC13472844; doi:10.3389/fphys.2026.1843298)
Supplement: Supplementary file 1 [file Supplementaryfile1.docx]

**SUPPLEMENTARY METHODS: DETAILED STATISTICAL ANALYSIS**

**Statistical environment and software**

All statistical analyses were performed in R using the RStudio 2025.09.2+418 “Cucumberleaf Sunflower” Release interface (Windows 11, 64-bit), with reproducible documents generated using Quarto 1.7.32. Data management and transformation were performed using base R functions and the tidyverse ecosystem.

The following R packages were used:

*readxl*: data import from Excel files.

*dplyr*, *tidyr*, *purrr*, and the tidyverse metapackage: data cleaning, transformation, restructuring (long/wide format), and generation of genetic model variables.

*logistf*: Firth’s penalized logistic regression for all genetic association analyses.

*HardyWeinberg*: exact test for Hardy–Weinberg equilibrium.

*genetics*: linkage disequilibrium (LD) estimation (D, D′, r, r²) and associated significance testing.

*haplo.stats*: haplotype reconstruction (*haplo.em*) and association testing (*haplo.score*).

*pwr*: power analysis (*pwr.2p2n.test*, *ES.h*).

P-values derived from homogeneous families of hypotheses were adjusted using the Benjamini–Hochberg false discovery rate (FDR) method (*p.adjust*), applied separately according to the type of analysis.

**Genotype processing and variable construction**

Five SNVs were analyzed: rs2298383, rs4822489, rs2236624, rs17650937, and rs8192446.

Genotypes were harmonized (e.g., AC ≡ CA) to ensure consistent coding. The minor allele was defined based on the full study population.

From these data, the following variables were constructed:

1. Global genotype distributions (2×k contingency tables)
2. Genotype-specific variables (presence vs absence of each genotype; 2×2 tables)
3. Allelic variables (minor vs major allele)
4. Genetic model variables:
   a. Dominant (≥1 copy of minor allele).
   b. Recessive (minor homozygote).
   c. Codominant (genotype as categorical variable).
   d. Additive (0, 1, or 2 copies of the minor allele).

**Genotype-based association analyses**

***Global genotype comparisons***

For each SNV, global differences in genotype distribution between groups were assessed using Fisher’s exact test (*fisher.test*) applied to 2×k contingency tables.

Five p-values (one per SNV) were obtained and adjusted using the Benjamini–Hochberg method within this family of hypotheses.

***Genotype-specific associations***

Within each SNV, genotype-specific associations were evaluated using Fisher’s exact test in 2×2 tables comparing the presence versus absence of each genotype.

P-values were adjusted using the Benjamini–Hochberg method within each SNV, treating each locus as an independent family of hypotheses.

***Allelic association analysis***

Allelic associations were assessed by decomposing genotypes into individual alleles and comparing minor versus major allele frequencies between groups using Fisher’s exact test.

P-values were adjusted across SNVs using the Benjamini–Hochberg method, as these tests represent a homogeneous family of hypotheses.

**Regression analyses under genetic models**

Associations between each SNV and preeclampsia were estimated using Firth’s penalized logistic regression (*logistf*), which is appropriate for sparse data and low-frequency genotypes.

Each SNV was analyzed under four genetic models:

1. Dominant.
2. Recessive (when minor homozygotes were present).
3. Codominant.
4. Additive.

Models were adjusted for clinically relevant covariates, including body mass index; previous pregnancies; previous deliveries; previous cesarean sections; previous abortions; gestational diabetes.

Covariates lacking variability within a given subset were excluded to ensure model stability.

Odds ratios (OR), 95% confidence intervals, and p-values were derived from the fitted models.

P-values were adjusted using the Benjamini–Hochberg method within each genetic model family.

**Stratified analysis by disease severity**

To evaluate associations across clinical subtypes, PE was recoded into two categories: LOPE, and EOPE.

For rs17650937 and rs8192446, dominant-model Firth regression was used to evaluate:

1. LOPE vs control.
2. EOPE vs control.
3. EOPE vs LOPE.

Covariates were included adaptively based on variability within each comparison subset.

No multiple-testing correction was applied to these analyses, as they correspond to a limited set of pre-specified clinical contrasts.

**Hardy–Weinberg equilibrium**

Hardy–Weinberg equilibrium (HWE) was assessed in the control group using the exact test (*HWExact*) implemented in the *HardyWeinberg* package.

Genotype counts were reconstructed after harmonizing genotype coding. This analysis was used as a quality-control step and interpreted descriptively.

**Linkage disequilibrium analysis**

Linkage disequilibrium between rs17650937 and rs8192446 was evaluated using D, D′, r, and r² metrics (*LD* function, *genetics* package).

Analyses were performed in controls and in the full sample.

**Haplotype analysis**

Haplotype inference was performed using the expectation–maximization algorithm (*haplo.em*, *haplo.stats* package), including individuals with complete genotype data for both SNVs.

Associations between haplotypes and preeclampsia were assessed using *haplo.score* under a binomial additive model.

P-values were adjusted using the Benjamini–Hochberg method within the haplotype family.

**Power analysis**

Power analyses were conducted using the *pwr* package.

Two scenarios were evaluated:

1. Using a very low population minor allele frequency (~0.0047), as suggested by a reviewer, resulting in very low statistical power.
2. Using observed allele frequencies in the study cohort, which yielded substantially higher power consistent with the detected effect.

**Multiple testing correction strategy**

To control the false discovery rate, p-values were adjusted using the Benjamini–Hochberg method within predefined families of homogeneous hypotheses:

1. Global genotype comparisons across SNVs.
2. Genotype-specific comparisons within each SNV.
3. Allelic analyses across SNVs.
4. Regression models within each genetic model family.
5. Haplotype-based associations.

This strategy preserves statistical power while avoiding over-conservative correction across biologically unrelated analyses.
